# A Sequential Markov Probabilistic Aggregation Algorithm for Causal Emergence in Markov Aggregation

**DOI:** 10.3390/e28080872

**Published:** 2026-08-03

**Authors:** Zhenjie Hou, Xuchu Dai

**Affiliations:** The Key Laboratory of Wireless Optical Communication, Chinese Academy of Sciences, School of Information Science and Technology, University of Science and Technology of China, Hefei 230026, China; daixc@ustc.edu.cn

**Keywords:** causal emergence, effective information, transition probability matrix, Markov aggregation

## Abstract

Causal emergence (CE) is a phenomenon in which macrodynamics provide better effective information (EI) than microdynamics. The CE is widely used as the objective function in Markov aggregation. The existing works focus on deterministic aggregation, which may not offer a good solution since the search space of each step is finite. To solve this problem, we propose a sequential Markov probabilistic aggregation (SMPA) algorithm. We first express the aggregation problem as an optimization problem, then find that the EI is maximized when the transition probability matrix is a permutation matrix, and prove that the optimization problem is a nonconvex function of the probabilistic aggregation matrix. In the SMPA algorithm, the optimization problem is split into multiple univariate optimizations. Compared with the deterministic aggregation algorithm, SMPA can achieve better greedy solutions. The experimental results indicate that probabilistic aggregation generally performs better than deterministic aggregation.

## 1. Introduction

Causal models represent the influence of a system’s subparts on other subparts or over the system as a whole and are ubiquitous in many areas, such as Markov chains [1,2,3], directed graphs (also called causal Bayesian networks) [4,5,6,7], and networks of interconnected mechanisms [8,9].

Causality refers to the connection between a cause and its resulting effect [10,11,12], which can reflect the structural characteristics of causal models. It describes the phenomenon in which an event, known as the cause, leads to another event, known as the effect [13,14,15]. A useful and flexible measure to assess the causality of causal models is effective information (EI) [16], which assesses the causal influence of one subset of a system on another [17]. The subsets of a system refer to the state of the Markov chain in the preceding and following moment, and a certain probability relationship exists between these states, and EI is the average of how much each state reduces the uncertainty about the future of the system. The EI can quantify the extent of causal influence within Markov chains and enable comparisons of values across different scales [18]. EI can be stated in a very general way, which is the mutual information between a set of interventions and their effects [19]. This is important because of mutual information’s understandability and its role as the centerpiece of information theory.

Causal emergence (CE) is defined as the difference in the EI values between the macro level and the micro level [20,21,22]. Several examples of discrete Markov chains [23] have been shown to exhibit causal emergence if their EI values of macro-level dynamics are greater than those of micro-level dynamics. Measures of EI and CE for dynamic innovation systems with continuous variables and complex networks are given in [24,25], respectively. Other measures are also possible for quantifying causal effects; Ref. [26] concluded that the CE is independent of the selection of the measure; thus, we focus only on measurement with the EI. The landscape of causal inference has evolved dramatically in recent years, moving from theoretical foundations to powerful applied frameworks. The authors of [27] proposed a novel idea to find a causal partitioning through edge-cutting in an adjoint graph, proving that the minimizer of the edge-cut ratio problem on an adjoint graph corresponds to a causal partitioning with a smaller vertex-cut ratio on G and non-violation of d-separation. Based on deep reinforcement learning and causal intervention, Causal Intervention Visual Navigation was proposed in [28], in which causal intervention was realized using front-door adjustment, as most confounders are hard to model explicitly.

According to the mapping relationship between fine-grained states and coarse-grained states, the methods of coarse graining can be divided into two categories. One is deterministic coarse graining, which is a fine-to-coarse, many-to-one map. Furthermore, the mapping from the macro level (coarse-grained state) to the micro level (fine-grained state) is a many-to-many map. The other is probabilistic coarse graining, which is a many-to-many map. Specifically, it focuses on coarse-to-fine maps, and the mapping from the micro level to the macro level is also a many-to-many map. Moreover, probabilistic coarse graining can be seen as a generative probabilistic model for a fine-grained system [29], which has a wide range of application scenarios, such as the full fine-grained system’s reconstruction [30] and probabilistic predictive estimates [31].

Generally, in probabilistic coarse graining, the coarse-grained states are seen as the latent variables, and the probabilistic models of fine-grained and coarse-grained need to be known. On the other hand, the construction of deterministic coarse graining is based on physical insight and localized lumping of several fine-grained states into a coarse-grained state, which is too restrictive to be applicable in many problems of interest without prior information.

There are two main methods for solving this problem to find the maximum value of the CE. A streamlined and potent aggregation method for general Markov chains, employing singular value decomposition (SVD) of the transition probability matrix (TPM), was proposed in [32]. The results are deterministic aggregation, and the values of the CE are actually not used in this method, which means that the implementation is independent of the specific value of the CE. The sequential generalized information-theoretic Markov aggregation (SGITMA) algorithm was proposed in [33], which is applicable for finding a deterministic aggregation matrix. To obtain a good solution, Ref. [34] runs the algorithm with many initializations to choose the best result. However, the optimal solution to the maximum value of the CE is not deterministic aggregation, and the search space of deterministic aggregation is finite, which indicates that the deterministic aggregation algorithms easily become stuck in local optima.

The meaning of causal emergence under the Markov approximation implies that as we coarse-grain microscopic states, the amount of EI transmitted from the current state to the next state will increase, which indicates that the inherent strength of the causal effect is increased under the coarse-grained Markov model and the particular state can influence the future state of a system more effectively. Probabilistic coarse-graining can mitigate the restrictions of the physical insight and the prior information for fine-grained systems. Thus, probabilistic causal emergence may be convenient for optimizing the targeted function CE. In this paper, we expand deterministic mapping (fine-to-coarse mapping) to probabilistic mapping. This method is referred to as "probabilistic aggregation" in this paper. We concentrate on how to aggregate time-homogeneous, irreducible, aperiodic, discrete-time, finite-state Markov chains while keeping the CE at a maximum. The main contributions are as follows:

- The probabilistic aggregation problem is expressed as an optimization problem, which is proven to be a nonconvex function of the probabilistic aggregation matrix. Specifically, the sufficient and necessary condition that the TPM is a permutation matrix is derived for the maximum value of the EI; then, it is proven that there is no matrix that can make the aggregated TPM a permutation matrix, and the nonconvex property of the CE is proven.
- The sequential Markov probabilistic aggregation (SMPA) algorithm is proposed to solve the optimization problem. The optimization is first decomposed into multistep multivariate optimization, and then each multivariate optimization is transformed to multistep univariate optimization for the convenience of solving. The gradient descent method (GDM) is used to solve each univariate optimization, and the detailed gradient derivation formula is derived.

Notation: We denote the column vectors and matrices by bold lower-case and bold upper-case letters, e.g., a and A; the row vectors are represented by $a^{T}$, which is the transpose of a. $a_{j}$ denotes the j-th element of a, and $A_{i,j}$ refers to the (i,j)-th element of matrix A. The random variables are represented by upper-case letters, e.g., *X*. The lower-case letters *x* denote their specific value, and $Pr_{X}\left(x\right)$ is the probability when *X* takes a value of *x*.

## 2. Markov Model and Definition of CE

When the discrete-time Markov chain X is time-homogeneous, irreducible, and aperiodic (see [35] for terminology) and the state space is finite, we abbreviate it as X∼Mar(X,P,μ). X denotes the alphabet, and $P\in R^{N\times N}$ is the TPM. μ is the unique stationary distribution and satisfies

(1) $$\mu^{T}P=\mu^{T},\sum_{i\in X}\mu_{i}=1.$$

$W\in R^{N\times M}$ is the probabilistic aggregation matrix, which satisfies the following:

(2) $$W_{i,j^{\prime }}\in \left[0,1\right],\forall i,\sum_{j^{\prime }=1}^{M}W_{i,j^{\prime }}=1,\forall j^{\prime },\sum_{i=1}^{N}W_{i,j^{\prime }}>0.$$

Let Y be the stochastic process obtained by aggregating X through W. In fact, Y is a hidden Markov process, which may not have Markov properties. $X^{\prime }$ is the best Markov approximation in the sense of minimizing the Kullback–Leibler divergence rate between Y and $X^{\prime }$ [33]. Afterward, the expression of the TPM and the stationary distribution of $X^{\prime }$ can be obtained as follows:

(3a)P′=UPW(3b)μ′=WTμ

where $U=\mathrm{diag}{\left(\mu^{\prime }\right)}^{-1}W^{T}\mathrm{diag}\left(\mu \right)$.

For $X^{\prime }$, we consider the state at time t,t+1 as a random quantity $A_{t}^{\prime },B_{t+1}^{\prime }$, respectively. When $X^{\prime }$ is time-homogeneous, the subscript *t* can be omitted; thus, the random quantity can be written as $A^{\prime },B^{\prime }$ instead. EI is a scoring metric based on mutual information [23], which is defined as follows:

(4) $$\begin{array}{cc} EI(P^{\prime })= & I\left(B^{\prime };A^{\prime }|A^{\prime }∼U\right)\\ = & \sum_{i^{\prime }=1}^{M}\sum_{j^{\prime }=1}^{M}Pr\left(a_{i^{\prime }}^{\prime },b_{j^{\prime }}^{\prime }\right)\log Pr\left(b_{j^{\prime }}^{\prime }|a_{i^{\prime }}^{\prime }\right)-\sum_{j^{\prime }=1}^{M}Pr\left(b_{j^{\prime }}^{\prime }\right)\log Pr\left(b_{j^{\prime }}^{\prime }\right)\\ = & \sum_{i^{\prime }=1}^{M}\sum_{j^{\prime }=1}^{M}\frac{1}{M}P_{i^{\prime },j^{\prime }}^{\prime }\log P_{i^{\prime },j^{\prime }}^{\prime }-\sum_{j^{\prime }=1}^{M}{{\bar{p}}_{j^{\prime }}}^{\prime }\log{\bar{p}}_{j^{\prime }}^{\prime }, \end{array}$$

where $A^{\prime }∼U$ denotes that the probability distribution of $A^{\prime }$ is uniform, *M* is the state number of $X^{\prime }$, and ${\bar{p}}^{\prime }$ is the average of the rows for $P^{\prime }$,

(5) $${\bar{p}}_{j}^{\prime }=\frac{1}{M}\sum_{i^{\prime }=1}^{M}P_{i^{\prime },j^{\prime }}^{\prime }.$$

Obviously, the mapping relationship between macro-level states and micro-level states is a many-to-many probabilistic mapping, which can easily be derived by Bayes’s formula. An aggregation strategy is a function that maps a set of microstates into macrostates, allowing for the derivation of a new dynamical model. CE occurs when the aggregated TPM possesses a larger EI than the original TPM [32], which is written as follows:

(6) $$CE\left(P,W\right)=EI\left(P^{\prime }\right)-EI\left(P\right).$$

The aggregation strategy can be expressed as the following optimization problem.

**Problem** **1.** *Letting X∼Mar(X,P,μ), we aim to maximize the CE by choosing W as an optimizer,*

(7) $$\underset{W}{max}CE\left(P,W\right),$$

*where optimization is over probabilistic mappings $X\to X^{\prime }$.*

## 3. The Lemmas of EI and CE

This section first gives the sufficient and necessary condition where the EI is maximized in Lemma 1; then, Lemma 2 explains why we cannot solve Problem 1 directly; finally, Lemma 3 proves that the CE is a nonconvex function of W.

**Lemma** **1.** *The permutation matrix $P^{\prime }$ is the sufficient and necessary condition for the maximum value of $EI(P^{\prime })$.*

The proof is given in Appendix A.

CE is a function of W; since EI(P) is a constant value, there is a fixed difference between $EI(P^{\prime })$ and CE(P,W). Although we obtain the structure of $P^{\prime }$ where CE(P,W) is maximum, Problem 1 is still difficult to solve, as we cannot find a W whose corresponding $P^{\prime }$ is the permutation matrix; the details are given in Lemma 2.

**Lemma** **2.** *Let X∼Mar(X,P,μ), where X is time-homogeneous, irreducible and aperiodic; for any probabilistic aggregation matrix W, $X^{\prime }$ is also a time-homogeneous, irreducible, aperiodic Markov chain, which can be written as $X^{\prime }∼Mar\left(X^{\prime },P^{\prime },\mu^{\prime }\right)$, and $P^{\prime }$ cannot be a permutation matrix.*

The proof of Lemma 2 is given in Appendix B.

Lemma 2 indicates that the aggregated Markov chain $X^{\prime }$ holds the properties of the original Markov chain X. In addition, $P^{\prime }$ cannot be a permutation matrix through the aggregation of X, which explains why we cannot obtain the maximum value of $EI(P^{\prime })$ directly.

**Lemma** **3.** *CE(P,W) is a nonconvex function of W.*

For the proof of Lemma 3, please see Appendix C.

As mentioned in Lemmas 1 and 2, EI is maximized if $P^{\prime }$ is a permutation matrix, but $P^{\prime }$ cannot be a permutation matrix in Markov aggregation. When searching for the mapping from the original state to the new cluster in the iteration of the SMPA algorithm, making the matrix as close as possible to the permutation matrix can be considered, which is likely to reduce the number of search dimensions during iteration. This property may improve the algorithm’s convergence, and will be studied more in future work. In addition, Lemma 3 shows that the optimization problem is nonconvex, which means that it is hard to find a global optimal solution. Hence, a suboptimal sequential iteration algorithm is utilized in Section 4.

## 4. The SMPA Algorithm

Although Problem 1 seems relatively simple in form, finding a good solution is difficult owing to its nonconvex property and requires tools for nonconvex optimization, such as simulated annealing or genetic algorithms. As mentioned in [33], this is still a difficult problem even for deterministic aggregation; thus, it is even more difficult for probabilistic aggregation.

The major problem is how to solve the probability values of W while keeping the row sum to unity. To solve this problem properly, we first decompose the optimization problem of variables W into the optimization of a specific row of W, and each optimization only changes the value of one row while keeping the others unchanged. In each optimization, the problem is a multivariate nonconvex optimization, which is still difficult to solve. For convenience, each multivariate optimization is then transformed into a multistep univariate optimization. In each of these, we regard only one value as an unknown quantity. Notably, normalization is required to ensure that the row sum is still unity and the values of the other rows in W remain unchanged.

In the SMPA algorithm, $iter_{max}$ denotes the maximum number of iterations. δ is the default value, which can accelerate the convergence of the algorithm. When the difference in the objective function between two adjacent iterations $\delta_{t}$ is less than the default value δ, the iteration ends prematurely. This means that the algorithm needs to meet two conditions to continue iterating; one is that the number of iterations should not exceed $iter_{max}$, and the other is $\delta_{t}\geq \delta$. $W_{init}$ is the initial aggregation matrix. If there are no requirements, it is randomly generated by sparse matrices. $W_{t}$ is the probabilistic aggregation matrix obtained in the *t*-th iteration, which is updated *N* times in one iteration and is consistent with the rows of the aggregation matrix. I,S,R are the iteration number, step size, and decay rate of the GDM, respectively, which have been presented in Table 1. $W_{t}^{\left(i,j^{\prime }\right)}$ denotes the aggregation matrix when the mapping from state *i* to cluster $j^{\prime }$ is considered in this iteration.

**Figure 1:**
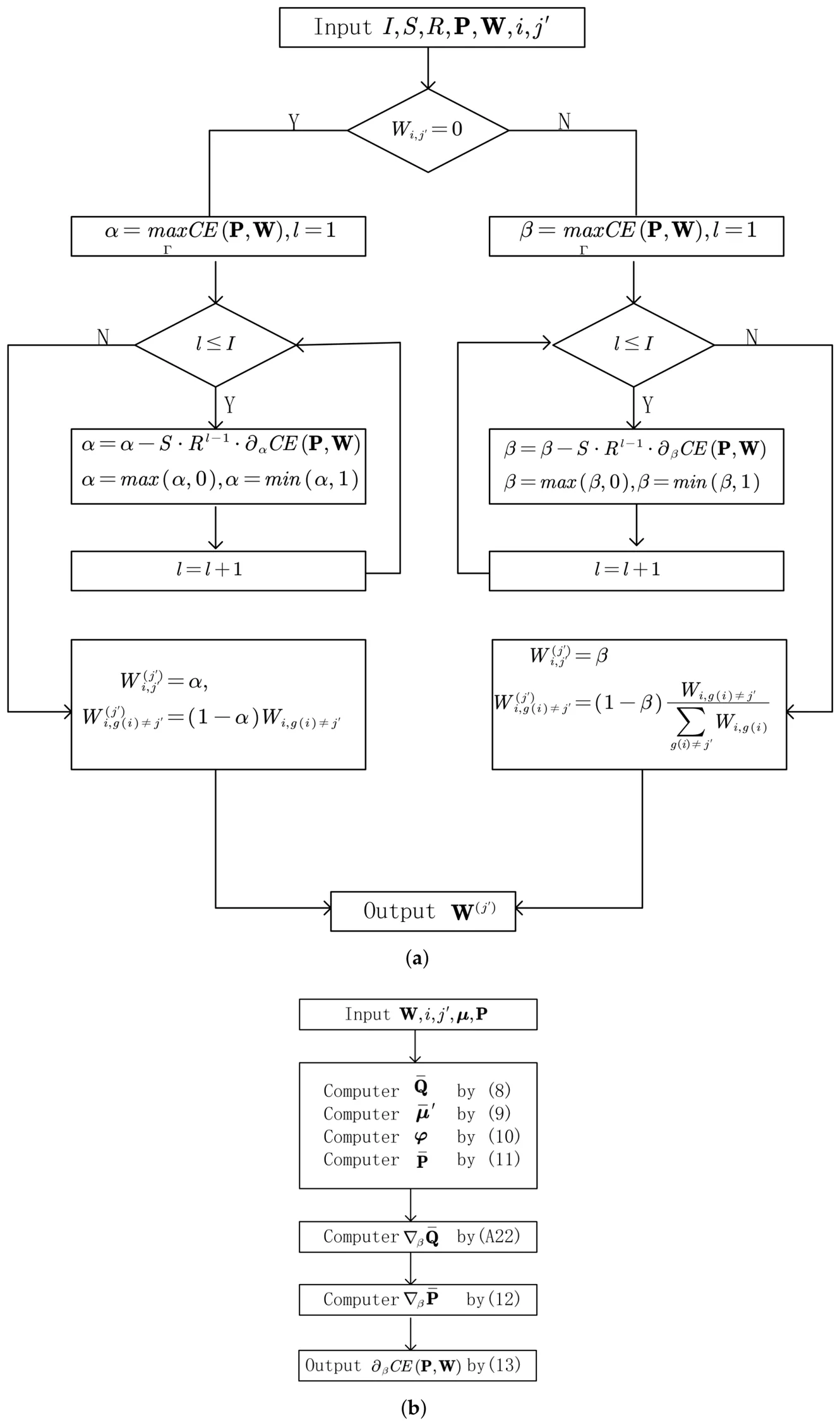
The flow chart of algorithms. (**a**) The flow chart of Algorithm 2. (**b**) The flow chart of Algorithm 3.

Here, we briefly explain the *t*-th iterative process of Algorithm 1: suppose that i∈X is mapped to cluster set $\left\{G_{t}^{i}\right\}$, which denotes the set mapped from the original state *i* in *t*-th iteration, and $W_{t}$ denotes the aggregation matrix in the *t*-th iteration. ${\bar{W}}_{t}$ is the intermediate variable obtained from Algorithm 2, and the ${\bar{W}}_{t}$ making the CE maximum is chosen as $W_{t}$. Since CE is a nonconvex function of ${\bar{W}}_{t}$, the optimal solution is difficult to find, and we use the GDM to find a suboptimal solution. The flow chart of GDM is given in Figure 1a.
**Algorithm 1** SMPA: Sequential Markov Probabilistic Aggregation**function** $W=SMPA(P,M,iter_{max},\delta ,I,S,R$, optional: $W_{init}$)     **if** $W_{init}$ is empty        $W_{0}\leftarrow$ Random aggregation matrix     **else**        $W_{0}\leftarrow W_{init}$     **end if**     t=0     **while** $t<iter_{max}$ and $\delta_{t}\geq \delta$ **do**        t=t+1, $W_{t}=W_{t-1}$        **for** i∈X            **for** $j^{\prime }\in X^{\prime }$               ${\bar{W}}_{t}=GDM(I,S,R$, $P,W_{t},i,j^{\prime })$            **end for**            $W_{t}=\underset{{\bar{W}}_{t}}{max}CE\left(P,{\bar{W}}_{t}\right)$        **end for**        $\delta_{t}=CE\left(P,W_{t}\right)-CE\left(P,W_{t-1}\right)$     **end while**     $W=W_{t}$

As shown in Algorithm 2, when $W^{\left(j^{\prime }\right)}$ is solved in each cycle, the normalization process is divided into two situations according to whether the mapping $W_{i,j^{\prime }}$ is zero in the previous iteration. The normalization of W for the two situations is different, and we use α,β for ease of distinction. The initial values of α,β are chosen from the set Γ={0.25,0.5,0.75}, which maximizes the CE. The purpose of Γ is to find a better initial point for the current iteration that can avoid getting stuck in poor local solutions. In theory, the larger the set, the better the algorithm’s performance, but it comes with an increase in computational complexity. Therefore, a compromise needs to be made between the performance and complexity. The deterministic aggregation algorithm takes values on zero and one. In order to distinguish the iteration process of SMPA and SGITMA, the intermediate value in the interval [0,1] is taken as the set Γ. α=max(α,0),α=min(α,1) is used to ensure that α∈[0,1]. Afterward, it is easy to update $W^{\left(j^{\prime }\right)}$ via simple computations. $\partial_{\alpha }CE\left(P,W\right)$ and $\partial_{\beta }CE\left(P,W\right)$ are the gradients of the objective function, which can be computed directly before the iteration of the algorithm since the function has only an unknown quantity α or β. The complete calculation processes of the gradient are given in Algorithm 3, and the flow chart is given in Figure 1b.
**Algorithm 2** GDM: Gradient Descent Method**function** $W^{\left(j^{\prime }\right)}=GDM(I,S,R$, $P,W,i,j^{\prime })$     **if** $W_{i,j^{\prime }}=0$        $\alpha =\underset{\Gamma }{max}CE\left(P,W\right)$        **for** l=1,⋯,I            $\alpha =\alpha +S\cdot R^{l-1}\cdot \partial_{\alpha }CE\left(P,W\right)$            α=max(α,0),α=min(α,1)        **end for**        $W_{i,j^{\prime }}^{\left(j^{\prime }\right)}=\alpha ,\;\;W_{i,g\left(i\right)\neq j^{\prime }}^{\left(j^{\prime }\right)}=\left(1-\alpha \right)W_{i,g\left(i\right)\neq j^{\prime }}\;\;$     **else**        $\beta =\underset{\Gamma }{max}CE\left(P,W\right)$        **for** l=1,⋯,I            $\beta =\beta +S\cdot R^{l-1}\cdot \partial_{\beta }CE\left(P,W\right)$            β=max(β,0),β=min(β,1)        **end for**        $W_{i,j^{\prime }}^{\left(j^{\prime }\right)}=\beta \;\;$,        $W_{i,g\left(i\right)\neq j^{\prime }}^{\left(j^{\prime }\right)}=\left(1-\beta \right)\frac{W_{i,g\left(i\right)\neq j^{\prime }}}{\sum_{g\left(i\right)\neq j^{\prime }}W_{i,g(i)}}\;\;$     **end if**

**Algorithm 3** GRAD: Calculation of the Gradient for CE**function** $\partial_{\beta }CE\left(P,W\right)$ = GRAD(W, $i,j^{\prime }$, μ, P)


     From W, $i,j^{\prime }$ get $\bar{W}$


    
$\bar{Q}={\bar{W}}^{T}\mathrm{diag}\left(\mu \right)P\bar{W}$

(8)
    
${\bar{\mu }}^{\prime }={\bar{W}}^{T}\mu$
(9)

    
$\varphi ={\left[-\mu_{i}\frac{W_{i,1}}{1-W_{i,j^{\prime }}},\;\cdots ,\;-\mu_{i}\frac{W_{i,m^{\prime }-1}}{1-W_{i,j^{\prime }}},\;\mu_{i},\;-\mu_{i}\frac{W_{i,m^{\prime }+1}}{1-W_{i,j^{\prime }}},\;\cdots ,\;-\mu_{i}\frac{W_{i,M}}{1-W_{i,j^{\prime }}}\right]}^{T}$
(10)

    
$\bar{P}=\mathrm{diag}{\left(\mu^{\prime }\right)}^{-1}W^{T}\mathrm{diag}\left(\mu \right)P\bar{W}$
(11)

     Compute $\nabla_{\beta }\bar{Q}$ by (A22)


     $\nabla_{\beta }\bar{P}=\mathrm{diag}{\left({\bar{\mu }}^{\prime }\right)}^{-2}\left\{\mathrm{diag}\left({\bar{\mu }}^{\prime }\right)\nabla_{\beta }\bar{Q}-\mathrm{diag}\left(\varphi \right)\bar{Q}\right\}$


     Output $\partial_{\beta }CE\left(P,W\right)$ by (13)



Let $i,j^{\prime }$ denote the specific row and column that we are concerned with in this cycle; $\bar{W}$ is the aggregation matrix after changing to the i-th row of the matrix W.

Then, the gradient of $\bar{P}$ can be derived as follows:

(12) $$\nabla_{\beta }\bar{P}=\mathrm{diag}{\left({\bar{\mu }}^{\prime }\right)}^{-2}\left\{\mathrm{diag}\left({\bar{\mu }}^{\prime }\right)\nabla_{\beta }\bar{Q}-\mathrm{diag}\left(\varphi \right)\bar{Q}\right\}$$

where the equation of $\nabla_{\beta }Q$ is given in Appendix D and $\bar{Q},{\bar{\mu }}^{\prime },\varphi ,\bar{P}$ are defined in (8), (9), (10) and (11), respectively.

The expression of $\partial_{\beta }CE\left(P,W\right)$ is as follows:

(13) $$\begin{array}{cc} \partial_{\beta }CE\left(P,W\right) & =\frac{1}{M}\sum_{m^{\prime }=1}^{M}\sum_{n^{\prime }=1}^{M}\nabla_{\beta }{\bar{P}}_{m^{\prime },n^{\prime }}^{\prime }\left(1+\log{\bar{P}}_{m^{\prime },n^{\prime }}^{\prime }\right)\\ \; & -\sum_{n^{\prime }=1}^{M}\left\{\frac{1}{M}\sum_{m^{\prime }=1}^{M}\nabla_{\beta }{\bar{P}}_{m^{\prime },n^{\prime }}^{\prime }\right\}\left\{1+\log\left(\frac{1}{M}\sum_{m^{\prime }=1}^{M}{\bar{P}}_{m^{\prime },n^{\prime }}^{\prime }\right)\right\} \end{array}$$

The detailed derivation of (13) is provided in Appendix D. Notably, $\partial_{\alpha }CE\left(P,W\right)$ is the special case of $\partial_{\beta }CE\left(P,W\right)$ when $W_{i,j^{\prime }}=0$; therefore, the calculation of $\partial_{\alpha }CE\left(P,W\right)$ can also be obtained from (13).

The calculation of $P^{\prime }$ requires $O\left(N^{2}M\right)$ multiplication operations, so the computational complexity of CE(P,W) is $O\left(N^{2}M\right)$. Since each iteration of the SGITMA algorithm requires NM calculations of the CE(P,W), the SGITMA has a computational complexity of $O\left(N^{3}M^{2}\right)$ per iteration.

The computational complexity of SMPA (Algorithm 1) per iteration is as follows. The computational complexity of $\bar{Q},\nabla_{\beta }\bar{P}$ is $O\left(N^{2}M\right)$, $O\left(N^{2}\right)$, respectively, and that of $\partial_{\beta }CE\left(P,W\right)$ is $O\left(N^{2}M\right)$; therefore, the computational complexity of Algorithm 3 is $O\left(N^{2}M\right)$. To compute Algorithm 2, Algorithm 3 must be called *I* times, so the computational complexity of Algorithm 2 is $O\left(IN^{2}M\right)$. Since each iteration of the SMPA algorithm requires calling Algorithm 2 NM times, SMPA has a computational complexity of $O\left(IN^{3}M^{2}\right)$ per iteration. Therefore, the computational complexity of SMPA is larger than that of SGITMA and is related to the value of *I*.

From the iteration process of Algorithm 1, it can be seen that SMPA is a kind of sequential optimization algorithm. Both the SMPA and SGITMA algorithms are sequential iterations in essence, and the objective value of each iteration will be higher than the previous one. When considering the mapping from the certain original state to the new cluster, the mapping probability of the SGITMA algorithm is either zero or one; however, that of the SMPA algorithm is extended to any value on the [0,1] interval. Thus, when the initial point is the same and only one row of the aggregation matrix is changed, the SMPA algorithm has a higher probability of finding a better solution compared with the SGITMA algorithm. In this paper, we consider maximizing the value of CE without the prior information of the model, in which case probability aggregation may find a higher objective value.

Since the $W_{t}$ is the aggregation matrix making the $CE_{t}$ maximum in the *t*-th iteration, and the matrix corresponding to a larger value of the CE is preserved, the $CE_{t}$ will not be lower than the $CE_{t-1}$. Therefore, $CE_{t}$ will not be decreased with the increase in iteration number *t*. Furthermore, from (A8) and (6), $CE_{t}\leq \log\left(M\right)-EI\left(P\right)$, the sequence $CE_{t}$ is bounded and non-decreased. Thus, the convergence of the algorithm can be guaranteed, but it needs to be fully researched whether this algorithm converges to a local optimal solution. A good way to improve the performance is to run the algorithm for many random initial W values and choose the best solution, which is already used in [34] to improve the performance of the deterministic aggregation algorithm.

## 5. Experiments

This section presents the simulation results of SMPA and is divided into two parts: the experimental conditions are given in the first subsection, which includes the generation of the TPM and the selection of parameter values, and the results are provided in the subsequent subsection. All source codes that support the findings of this study are openly available at the GitHub repository: https://github.com/jchzj/SMPA (accessed on 21 May 2026).

### 5.1. Experimental Conditions

**Figure 2:**
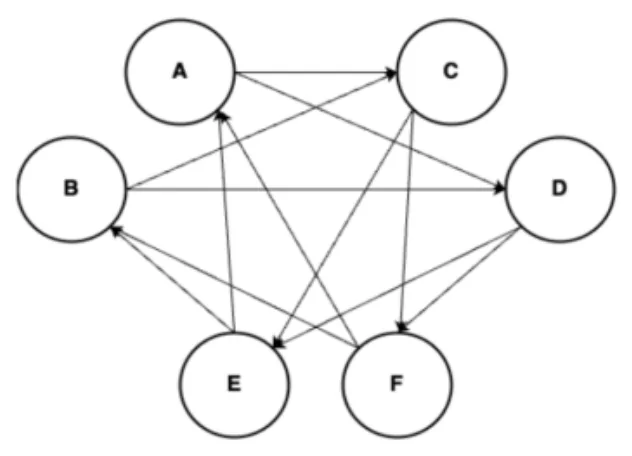
Boolean network model. The ABCDEF are six nodes, with each node taking a value of 0 or 1, and the arrows represent the connection relationships between nodes.

The Boolean network model with 6 nodes and 12 edges is shown in Figure 2. Each node has two possible values {0,1} and can only interact with its two network neighbors. The system consists of six binary elements, the set is {ABCDEF}, and each possible value is seen as a micro-mechanism, that is, an AND gate (two inputs and two outputs) operating over some intrinsic noise [32].

The node dynamics of Figure 2 are presented in Table 2. For any node, each row corresponds to the state combination of all the nodes’ neighbors in the previous time step, and each column is the probability of 0 or 1 of the node at the current time step. For example, if AB is 00 at time $t_{n}$, since CD interacts with AB only, then node *C* or *D* at time $t_{n+1}$ is 0 with a probability of 0.7 and is 1 with a probability of 0.3, which is the first row of Table 2. The probabilities of 00,01,10,11 for the values of CD are 0.49,0.21,0.21 and 0.09, respectively. The transition probabilities between AB and CD are presented in Table 3.

**Figure 3:**
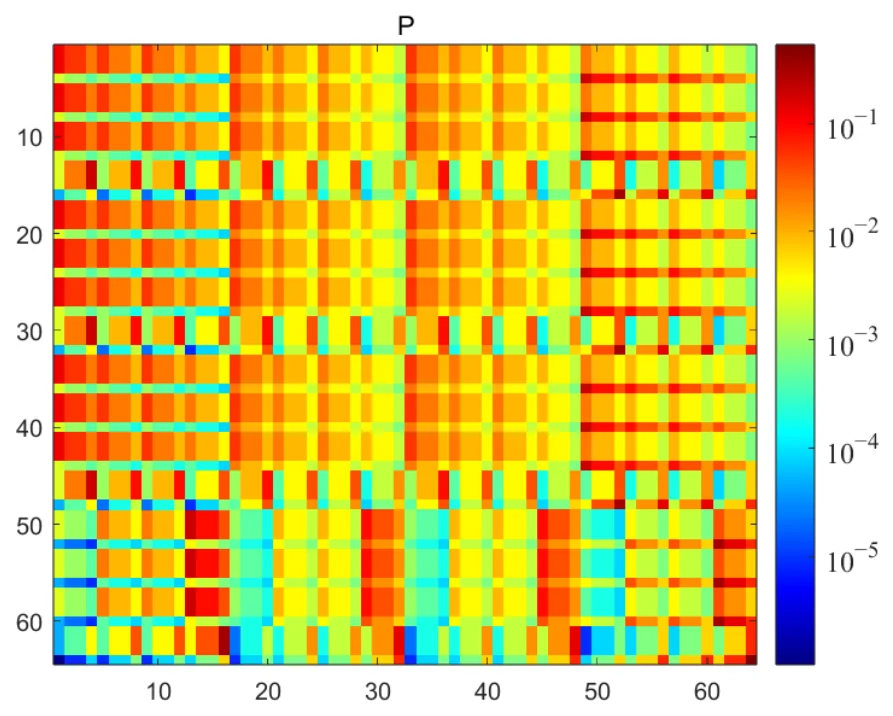
TPM of the Boolean network. The colors represent numerical values, and the darker colors represent larger values, which can be obtained from the Boolean network.

The 64×64 TPM is constructed by setting the system into all possible micro states from [000000] to [111111], and the values are presented in Figure 3.

From the TPM, it is easy to verify that X is time-homogeneous, irreducible, and aperiodic. In the following experiments, the SMPA is randomly initialized. *N* is set to 64, and we set $iter_{max}$, δ, *I*, *S*, and *R* to 50, 0.001, 10, 0.25, and 0.9, respectively, and the SMPA and SGITMA are repeated 50 times. Each iteration is initialized by a stochastic probabilistic aggregation matrix. All experiments were run in Matlab 2024a with CPU i7-6700 @3.4 GHz and 16 GB of RAM. In the experiments, the cost function of SGITMA and SMPA is CE with the optimization variable W, and the W corresponding to the maximum CE is the final result of the SMPA and SGITMA. For different values of *M*, we choose the case where the value of CE is the maximum to compare the iteration number. The simulated annealing (SA) algorithm is a probability-based global optimization algorithm that randomly searches for the global optimal solution of the objective function in the solution space, which has the chance to avoid getting stuck in local optimal solutions. The SA algorithm mainly includes three steps: neighborhood search, acceptance judgment, and cooling iteration. Neighborhood search refers to randomly generating a new solution near the current solution, and the difference dCE between the objective function of the new solution and the current solution is calculated. In acceptance judgment, the new solution is accepted directly if it is better; otherwise, it is retained with probability exp(T·dCE), where *T* is the parameter for controlling the acceptance probability. The cooling iteration means that as the number of iterations increases, the T value gradually decreases, allowing the smooth transition from global exploration to local convergence. Due to the practicality of the SA algorithm, this paper uses it as one of the benchmarks.

The performance improvement percentage (PIP) is calculated as follows:

(14) $$PIP=\left(CE_{SMPA}-CE_{SGITMA}\right)/CE_{SGITMA}*100,$$

where $CE_{SMPA},CE_{SGITMA}$ denote the maximum values of the CE obtained by the SMPA and the SGITMA, respectively.

### 5.2. Experimental Results

#### 5.2.1. *N* = 64

**Figure 4:**
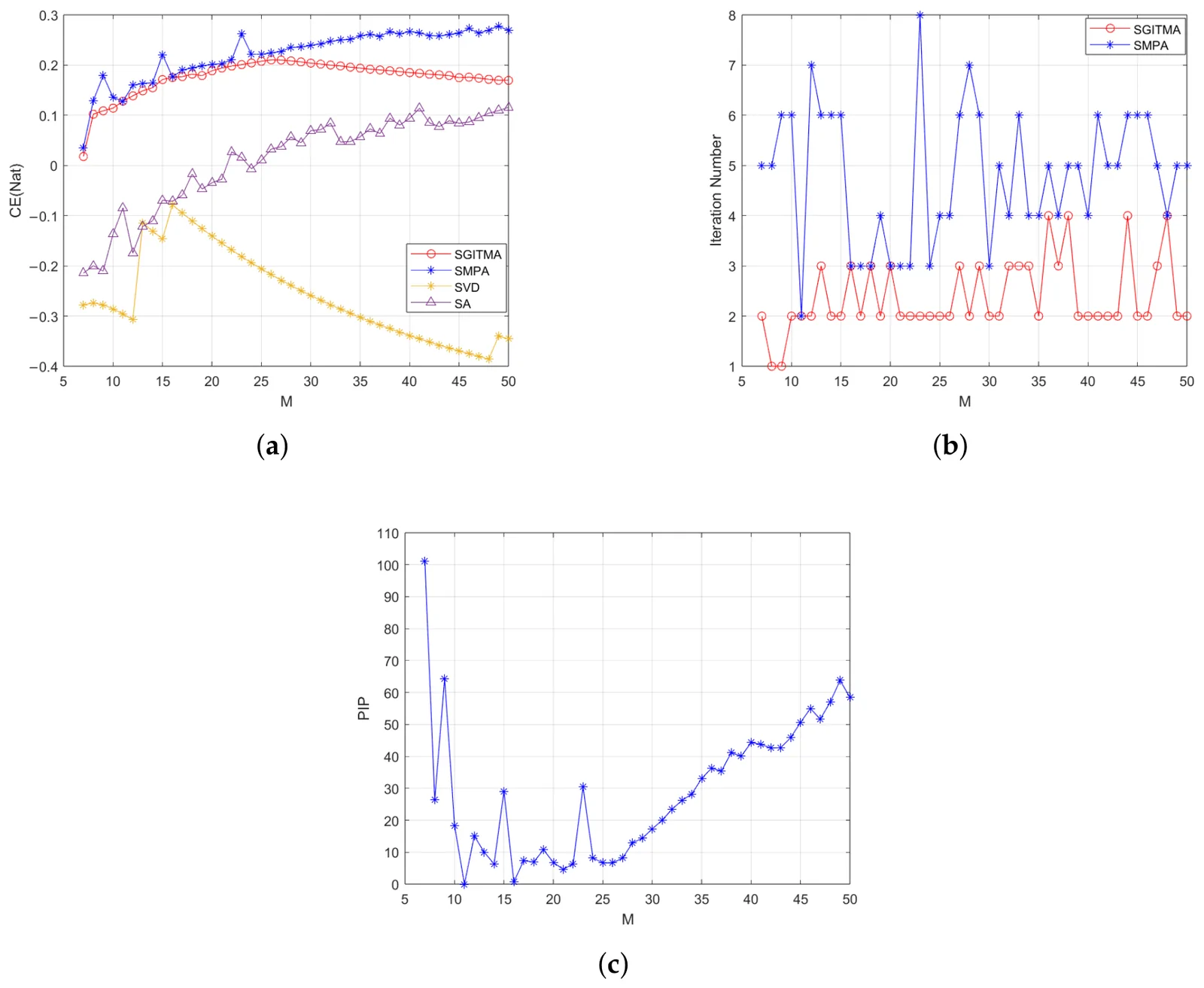
The experimental results of the SGITMA, SMPA and SVD for N=64 with 50 stochastic initializations. (**a**) Curves showing the value of the CE with increasing *M*. (**b**) The number of iterations required for algorithm convergence when the CE is at its maximum. (**c**) The PIP of the SMPA compared with that of the SGITMA.

An increasing trend of performance improvement with increasing *M* is shown in Figure 4a. With multiple iterations, the performance of the SMPA is better than that of the SGITMA. When *M* is small, the improvement in the SMPA is small except for certain points where the algorithm can escape the poor local optimal solution. The SMPA achieves significant improvement once *M* is greater than 25. However, when *M* increases to 25, the performance improvement of the SGITMA algorithm actually decreases because the original number of states *N* is a fixed value of 64: the larger the value of *M* is, the more original states will be mapped to a single cluster. When *M* is equal to *N*, the CE decreases to zero for deterministic aggregation; thus, the performance of SGITMA first increases but then decreases. Therefore, the performance gap between the two algorithms increases with increasing *M*. The SVD algorithm cannot achieve good performance since the specific value of the EI is not used in its implementation, and it is useful for dynamic reversibility. The experimental results of the simulated annealing (SA) method are also added in Figure 4a, and each experiment consists of 500N random changes to the rows of the mapping matrix. The performance of the SA algorithm is better than that of the SVD algorithm since the specific value of the EI is used in iteration when N=64, but it is worse than that of SMPA and SGITMA.

The number of iterations required for the convergence of the algorithms when the CE is at its maximum is shown in Figure 4b. The convergence speed of the SMPA is slower than that of the SGITMA algorithm, but the number of iterations does not significantly increase. A larger iteration number corresponds to greater performance improvement in general. When M=11 or 16, the performance of the two algorithms is the same, and the number of iterations required for convergence is also the same. For other values of *M*, the SMPA normally requires more iterations to achieve performance improvement since the search space is larger than that of the SGITMA at each step, thus requiring more iterations to reach a better local optimum. Additionally, the SMPA achieves better performance when the number of iterations does not increase under some circumstances, such as when M=20 or 48.

The PIP of the SMPA is shown in Figure 4c, which is consistent with expectations since deterministic aggregation is a special case of probabilistic aggregation and the search space of the SMPA is relatively large.

**Figure 5:**
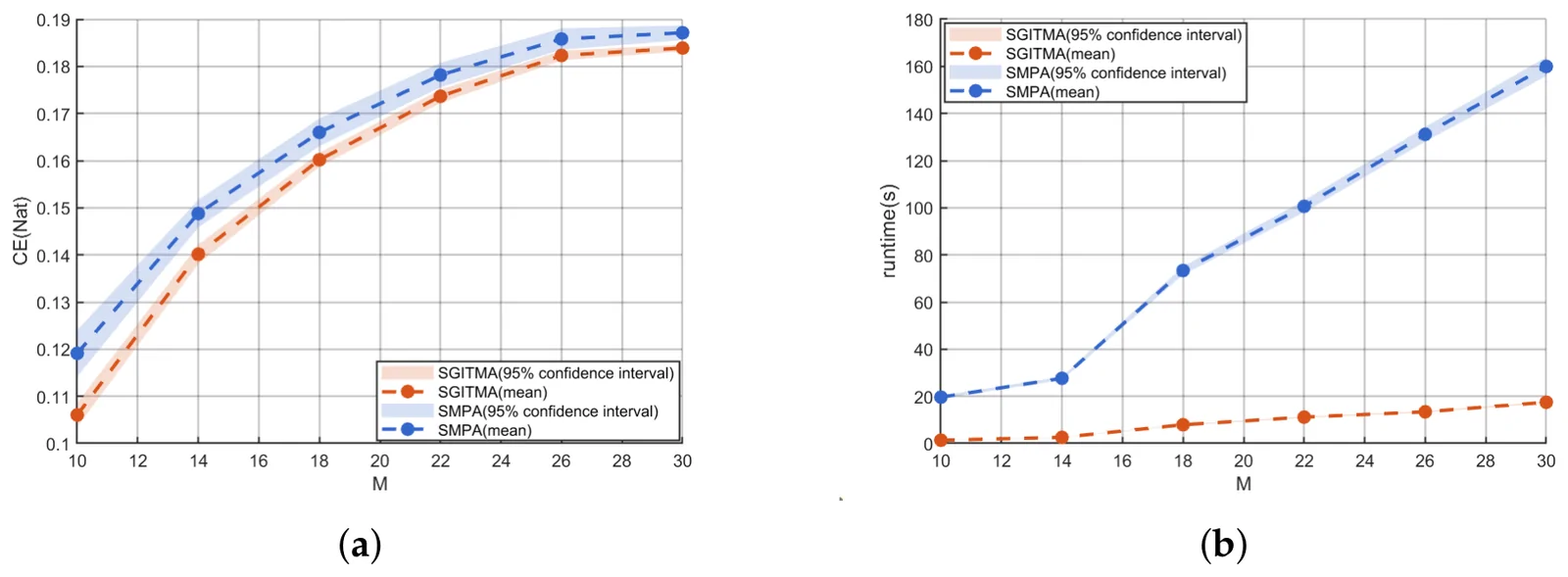
The experimental results of the SGITMA and SMPA for N=64 with 200 initializations. (**a**) The 95% confidence interval and the mean value of the CE for the SGITMA and SMPA. (**b**) The 95% confidence interval and the mean value of the runtime for the SGITMA and SMPA.

To check the confidence level of each iteration of the algorithm, 200 random initializations were performed, the results of which are shown in Figure 5a. The mean value and standard deviation of the runtime are given in Figure 5b. To check the confidence level of each iteration of the algorithm, 200 random initializations were performed, and the results of each iteration of the algorithm are shown in Figure 5a; the mean value and the standard deviation of the runtime are given in Figure 5b. Since the number of experiments is 200, which exceeds 30, the t-distribution is chosen to obtain the confidence intervals. For each *M*, the mean $CE_{mean}$ and standard deviation $CE_{std}$ of these 200 CE values can be calculated. The margin error (ME) for CE can be obtained by $ME=tinv\left(0.975,199\right)\times CE_{std}/\sqrt{200}$, where tinv(0.975,199) denotes the inverse cumulative distribution function of the t-distribution with cumulative probability 0.975 and degrees of freedom 199. Thus, the 95% confidence interval can be obtained by $CE_{mean}\pm ME$. Correspondingly, the same method can be used to obtain the 95% confidence interval and the mean value of the runtime for the SGITMA and SMPA.

As shown in Figure 5a, the mean value of the CE with 200 random initializations for the SMPA algorithm is larger than that of the SGITMA algorithm, which indicates that the overall performance of the former is better than that of the latter. The standard deviation of the SMPA algorithm is larger than that of SGITMA, so it needs to be initialized many times to find a good solution, and the standard deviation of both algorithms decreases as the value of *M* increases. The runtime refers to the required time from the start for the algorithm to converge under one initialization. Both the average runtime and the standard deviation of the SMPA algorithm are higher than that of the SGITMA algorithm, because the former generally requires more iterations to converge and the runtime is also related to the value of iteration number *I* in the GDM.

**Figure 6:**
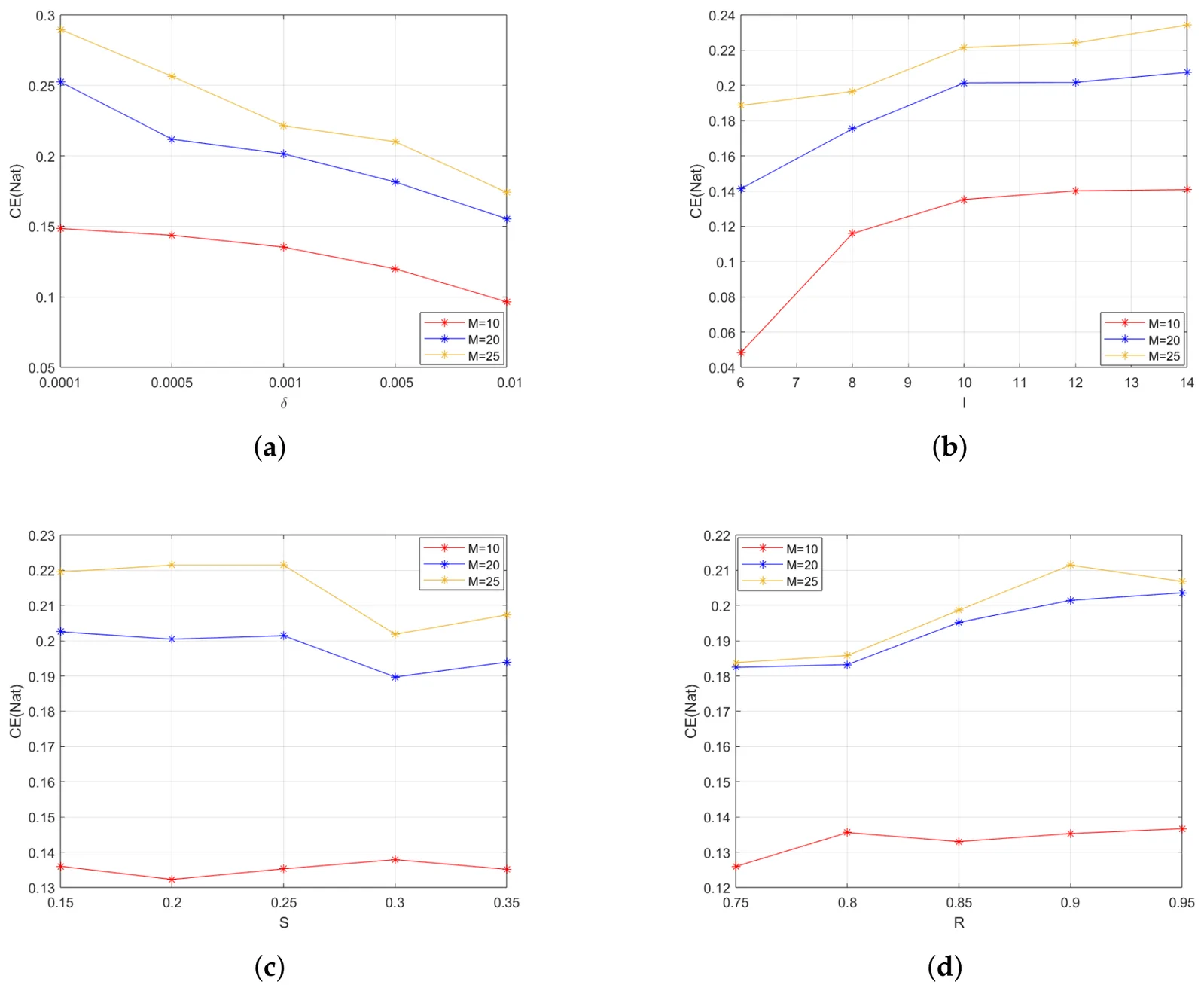
The influence of parameters δ,I,S,R for SMPA when N=64 and M=10,20,25 with 50 initializations. (**a**) The performance of the SMPA for δ=0.0001,0.0005,0.001,0.005,0.01. (**b**) The performance of the SMPA for I=6,8,10,12,14. (**c**) The performance of the SMPA for S=0.15,0.2,0.25,0.3,0.35. (**d**) The performance of the SMPA for R=0.75,0.8,0.85,0.9,0.95.

For a sensitivity analysis of the parameters, we conducted several experiments, the results of which are shown in Figure 6. In the experiments, δ, *I*, *S*, and *R* are set to 0.001, 10, 0.25, and 0.9, respectively. For each subgraph, we change only one parameter while keeping the other three unchanged to compare the impact of that parameter on algorithm performance.

As shown in Figure 6a, as the value of δ increases, the performance of the SMPA algorithm continues to decrease, because the iteration terminates prematurely for large values of δ. If the value of δ is too small, it will result in too many iterations and increase the algorithm’s runtime. It can be seen from Figure 6b that as the value of *I* increases, the performance of the algorithm continues to increase, which is the opposite of the influence of δ. That is, the more iterations there are, the better the performance of SMPA, which is consistent with the results of the theoretical analysis. When the value of *I* increases from 10 to 12, the performance improvement is relatively small, and the step size of the last few iterations in the GDM is very small. As shown in Figure 6c, when the value of *S* is small, the performance of the algorithm may improve slightly; however, for a large value of *S*, the algorithm may not converge to a good local optimal solution due to the excessively large step size. The experimental results show that the value of *S* can be selected within the range of 0.15 to 0.25. As shown in Figure 6d, when the value of *R* is less than 0.9, the performance of the SMPA algorithm may slightly improve for small values of *M*, but there will be a significant performance loss for large values of *M*, since the rapid decay of the step size weakens the performance of the last few iterations in the GDM. Therefore, when setting the parameters δ,I,S,R, both high and low parameter values can have negative effects. Based on experimental results, we choose intermediate values.

**Figure 7:**
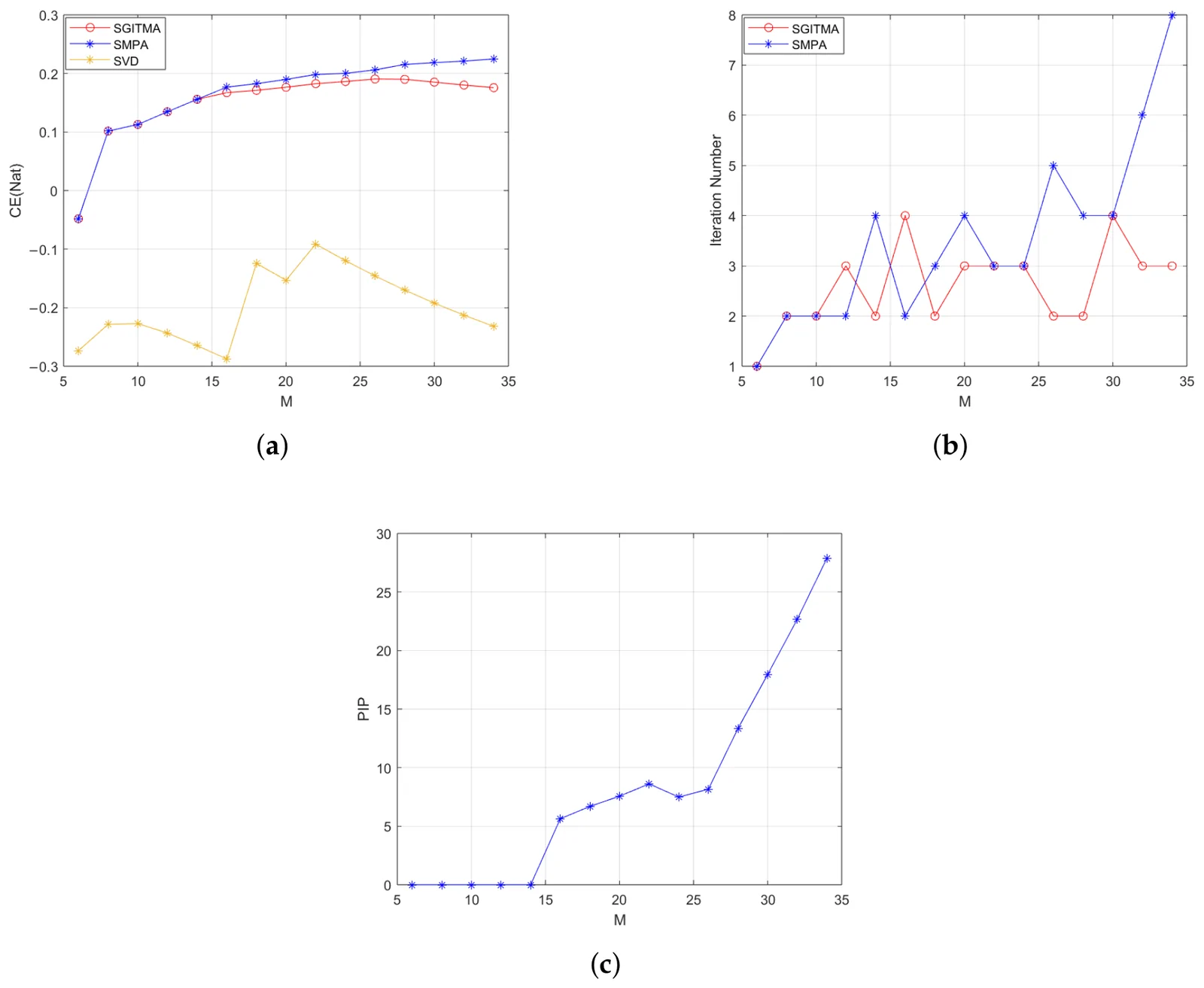
The experimental results of the SGITMA, SMPA and SVD for N=64 when the initialization of W is the first iteration result of the SGITMA with 50 initializations. (**a**) Curves showing the value of the CE with increasing *M*. (**b**) The number of iterations required for algorithm convergence when the CE is at its maximum. (**c**) Compared with that of the SGITMA, the PIP of the SMPA.

To observe the impact of initialization for W on algorithm performance, we also use the aggregation matrix obtained from the first iteration of SGITMA as the initialization. The experimental results are given in Figure 7.

As shown in Figure 7a, when the initialization of W is the first iteration result of SGITMA, the performance of the SMPA and SGITMA are almost the same if *M* is small. The SMPA significantly improves when *M* is greater than 15, and the changes in PIP are not significant compared with those in Figure 4c. That is, when *M* is small, the better initialization choice is the stochastic aggregation matrix; for a larger value of *M*, we can use the first iteration result of SGITMA as initialization to improve the convergence speed since the impact of performance is not large.

The number of iterations required for the algorithm convergence when the CE is at its maximum is shown in Figure 7b. Compared with Figure 4b, the number of iterations is reduced for most values of *M*, which sacrifices the algorithm performance in exchange for convergence speed.

**Figure 8:**
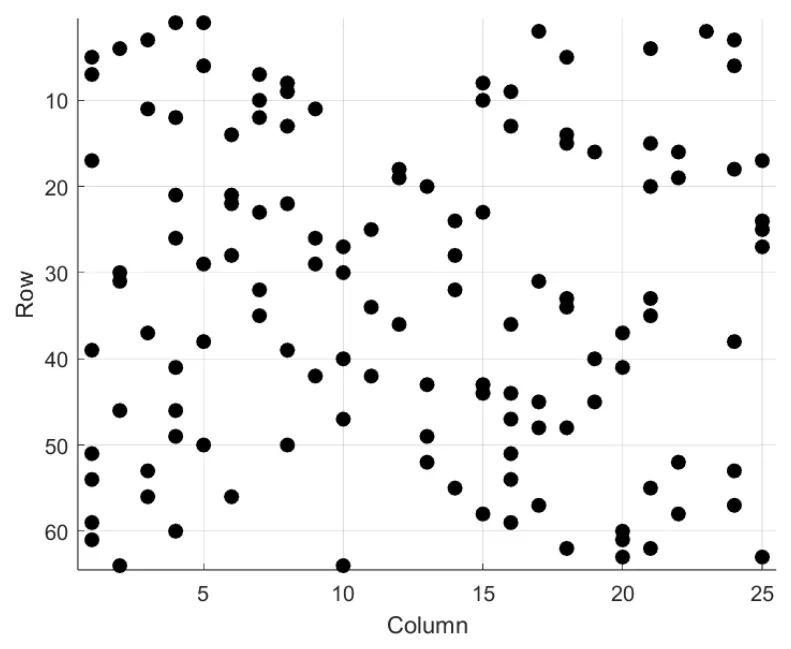
The aggregation matrix when N=64,M=25. The black dots indicate nonzero values in the matrix.

From the experimental results, we find that the structure of the learned aggregation matrix W is sparse; that is, the learned W has only a few nonzero values in each row. When the original state is mapped to fewer new states, it is more likely to obtain a larger value of CE. To better visualize this sparse feature, one instance of the aggregation matrix when N=64,M=25 is shown in the Figure 8. The proportion of nonzero elements is 8%, which means that the learned aggregation matrix is sparse. And how to utilize this sparse property of the learned W will be fully investigated in our future work.

#### 5.2.2. N=256

In this section, we compare the performance of algorithms under a large-scale Markov chain. *N* is set to 256, and we set $iter_{max}$, δ, *I*, *S*, and *R* to 50, 0.001, 10, 0.25, and 0.9, respectively. The SMPA and SGITMA are repeated 50 times.

**Figure 9:**
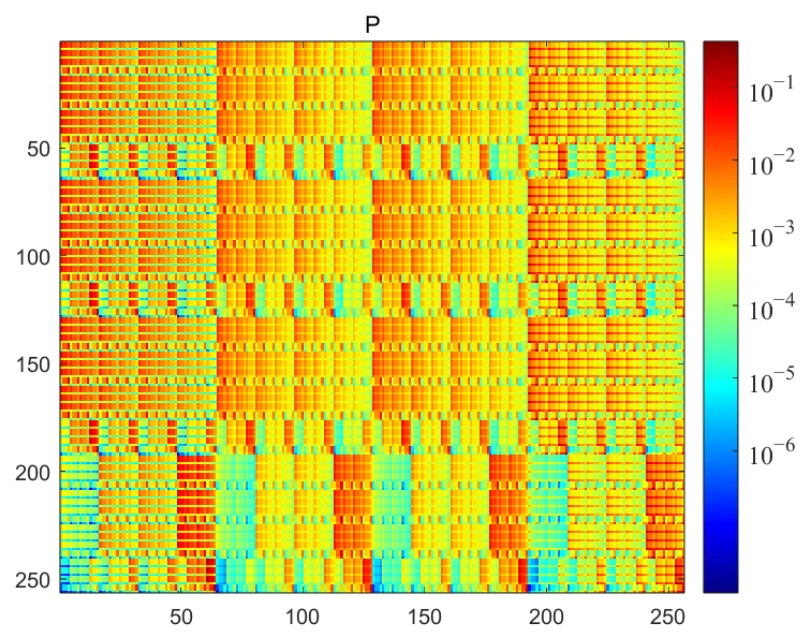
The TPM of a large Markov chain when N=256. The colors represent numerical values, and the darker colors represent larger values, which can be obtained directly from the Boolean network.

**Figure 10:**
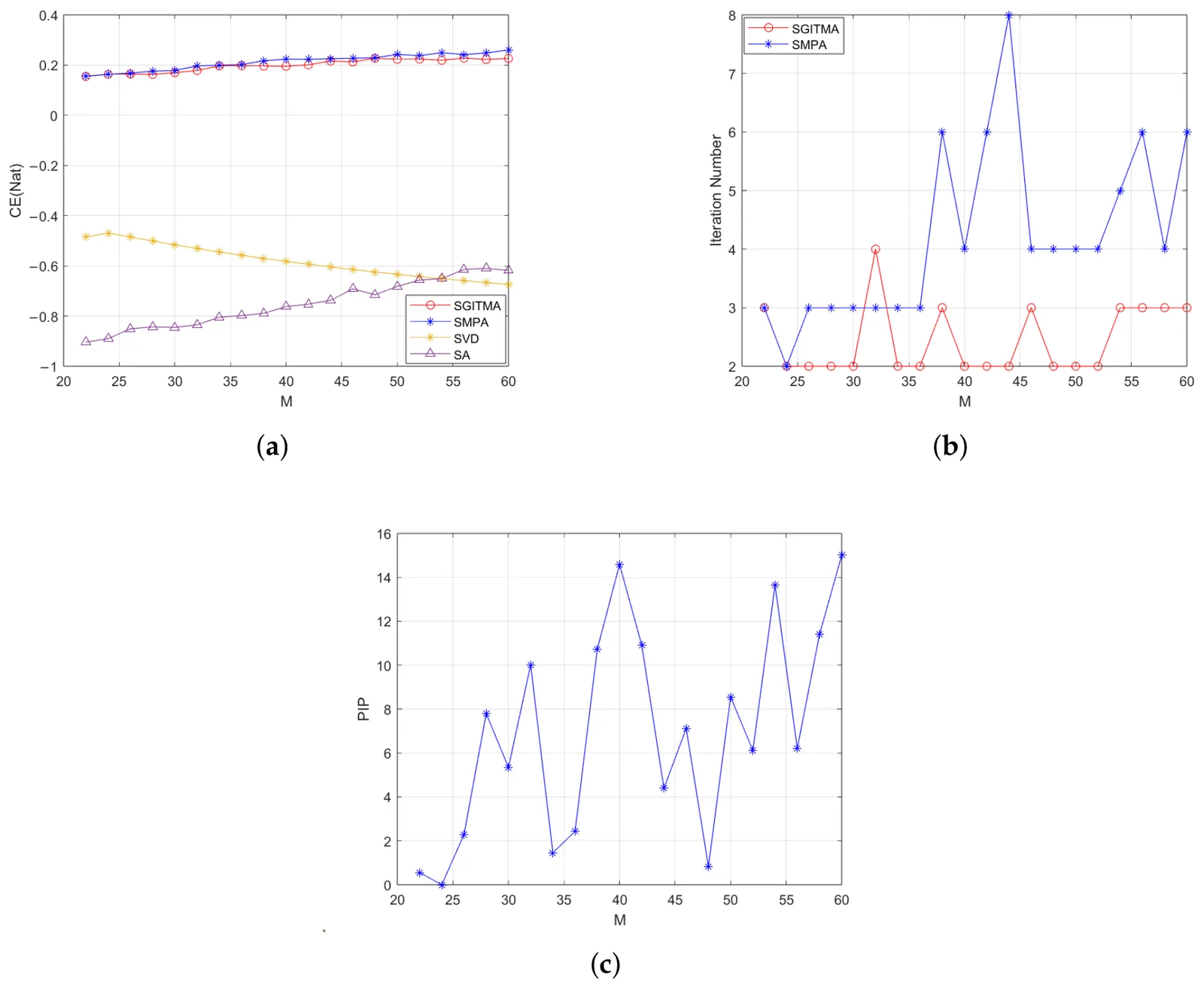
The experimental results of the SGITMA, SMPA and SVD for N=256 with 50 stochastic initializations. (**a**) Curves showing the value of the CE with increasing *M*. (**b**) The number of iterations required for algorithm convergence when the CE is at its maximum. (**c**) The PIP of the SMPA compared with that of the SGITMA.

**Figure 11:**
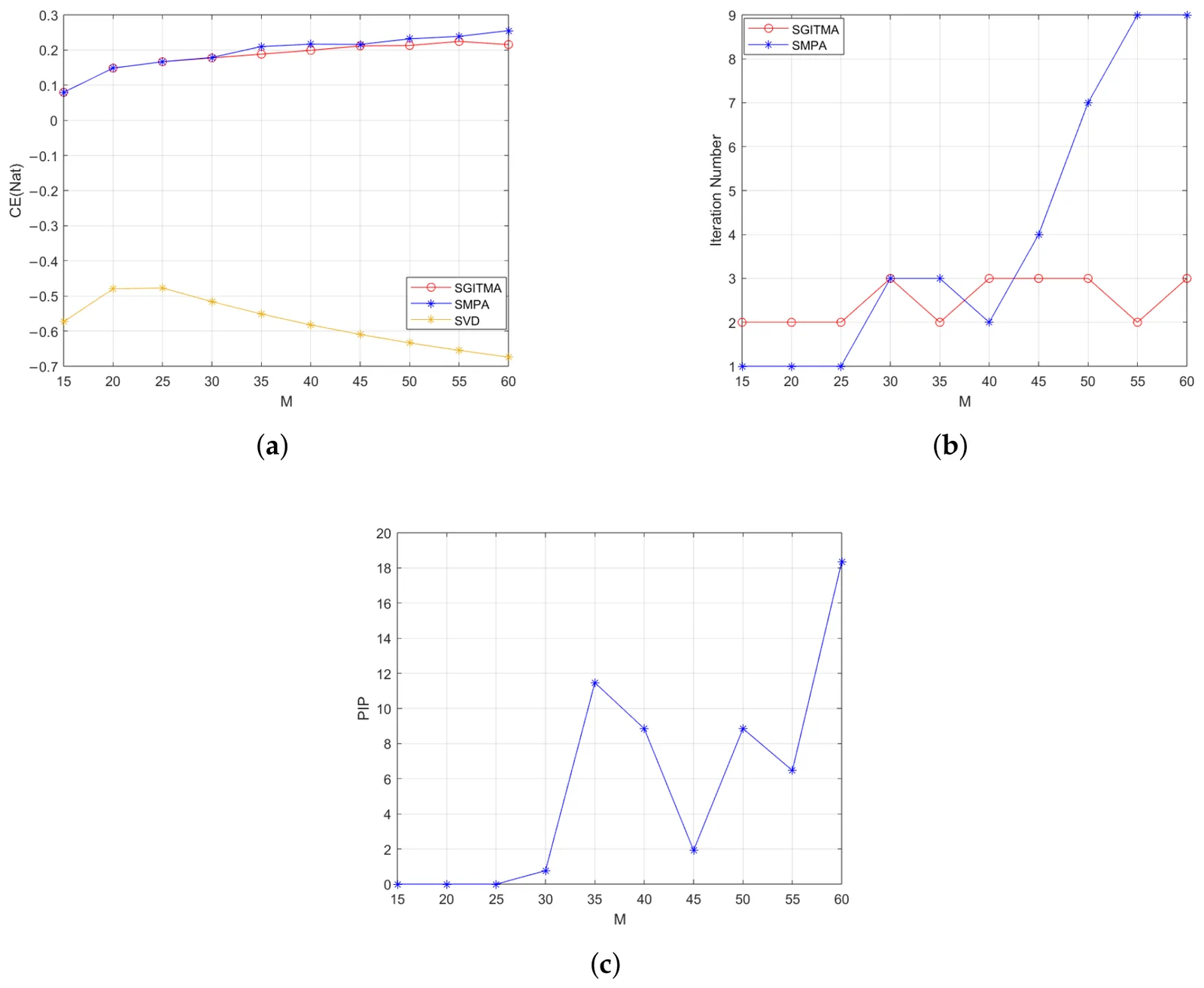
The experimental results of the SGITMA, SMPA and SVD when the initialization of W is the first iteration result of the SGITMA with 50 initializations. (**a**) Curves showing the value of the CE with increasing *M*. (**b**) The number of iterations required for algorithm convergence when the CE is at its maximum. (**c**) Compared with that of the SGITMA, the PIP of the SMPA.

The TPM is given in Figure 9, which shows that X is time-homogeneous, irreducible, and aperiodic. The experimental results corresponding to the two initialization strategies are shown in Figure 10 and Figure 11, respectively.

As shown in Figure 10a, when the initialization of W is a stochastic probabilistic aggregation matrix, SVD still performs poorly for large Markov chains since the algorithm does not use the specific value of the EI. The performance of the SMPA is still better than that of the SGITMA. The performance improvement of the SMPA is small when *M* is small but significant for high values of *M*, which is consistent with the result of the Markov chain when N=64. The number of iterations required for the convergence of the SMPA is still larger than that for the SGITMA; however, the number of iterations does not significantly increase. As *N* increases, more iterations are required to ensure the convergence of the algorithm in general.

Although the SMPA performs better than the SGITMA algorithm for bigger state spaces, its computational complexity—$O\left(IN^{3}M^{2}\right)$ per iteration—is significantly greater and increases linearly with $M^{2}$ and $N^{3}$. The methods to reduce the computational complexity will be studied in future work.

When the initialization of W is the first iteration result of SGITMA, as shown in Figure 11a, the performance of the SGITMA and SMPA are almost the same for small values of *M*, but the latter’s performance improves significantly for high values of *M*, which is consistent with the result of the Markov chain when N=64. If the initialization of W is closer to the local solution, the number of iterations required for convergence decreases for most values of *M*, especially for small values, thus sacrificing the performance of the SMPA algorithm in exchange for convergence speed.

## 6. Discussion

CE is an effective measure for assessing the improvement in causality between the macro and micro levels and can be used as the objective function of Markov aggregation. Previous works focused on deterministic aggregation, which may not find a good solution to the optimization problem since the search space in each step is finite. To obtain better solutions for each step, we consider probabilistic aggregation, which is more likely to escape from the local optimal solution. The SMPA algorithm was proposed to find the probabilistic aggregation matrix, which splits the optimization problem into multistep univariate optimizations. Compared with deterministic aggregation, the SMPA’s performance generally improves well.

Nevertheless, this work has several limitations. One of the major ones is the choice of a good initial point for the optimization problem. Deterministic and probabilistic aggregation are influenced by the initial point, and a good initial point can accelerate the convergence of the algorithm and improve its performance. More mathematical tools and methods should be developed to study this problem. Moreover, the order of optimization objectives in sequence optimization algorithms is a research hotspot, and accelerating the convergence speed of the algorithm by changing the order of the optimization objectives is also a worthwhile research question. Additionally, regardless of whether deterministic or probabilistic aggregation is used, the TPM of the original system cannot be fully recovered from that of the reduced system, since the aggregation from high to low dimensions results in information loss. The learned aggregation matrices are sparse, which is an interesting conclusion observed through experiments, and future work will consider utilizing sparsity as a feature to improve the convergence property.

The application of the theory of causal emergence to neuroscience may help solve longstanding problems involving scale, such as the debate over whether brain circuitry functions at the scale of neural ensembles or individual neurons [36,37,38]. An experimental way to resolve these debates is to systematically measure EI in ever-larger groups of neurons, eventually arriving at or approximating the cortical scale with EI. Furthermore, the SMPA algorithm can be used for pairwise clustering [39,40]. The cost function of pairwise clustering is based on maximizing the mutual information between consecutively visited clusters of states of the Markov chain defined by the similarity matrix; the only difference from our optimization problem is the objective function. Additionally, the problem of semi-supervised clustering [41,42] was connected to constrained Markov aggregation, for which there are more constraints than pairwise clustering, and the SMPA may be used to solve such clustering with some adjustments.

## Figures and Tables

**Table 1:** Notation table.

Symbol	The Meaning of the Symbol
X	the original Markov chain
P	the TPM of X
μ	the unique stationary distribution of X
W	the probabilistic aggregation matrix
$X^{\prime }$	the reduced Markov chain
$P^{\prime }$	the TPM of $X^{\prime }$
*N*	the number of states for X
*M*	the number of states for $X^{\prime }$
$\mu^{\prime }$	the unique stationary distribution of $X^{\prime }$
$iter_{max}$	the maximum iteration number
δ	the default value for accelerating the convergence
$\delta_{t}$	the difference in the objective function between the *t*-th and (t−1)-th iterations
*I*	the iteration number of the GDM
*S*	the step size of the GDM
*R*	the decay rate of the GDM
$W_{init}$	the initial aggregation matrix
$W_{t}$	the aggregation matrix in the *t*-th iteration
${\bar{W}}_{t}$	the intermediate variable obtained from Gradient Descent Method
$\left\{G_{t}^{i}\right\}$	the set mapped from the original state *i* in the *t*-th iteration

**Table 2:** Node dynamics of the Boolean network.

$t_{n}\setminus t_{n+1}$	0	1
00	0.7	0.3
01	0.7	0.3
10	0.7	0.3
11	0.1	0.9

**Table 3:** Transition probabilities between AB and CD.

AB∖CD	00	01	10	11
00	0.49	0.21	0.21	0.09
01	0.49	0.21	0.21	0.09
10	0.49	0.21	0.21	0.09
11	0.01	0.09	0.09	0.81

## Data Availability

The data are contained within the article. The original contributions presented in this study are included in the article. Further inquiries can be directed to the corresponding author.

## Abbreviations

The following abbreviations are used in this manuscript:

CE	causal emergence
EI	effective information
SMPA	sequential Markov probabilistic aggregation
SVD	singular value decomposition
TPM	transition probability matrix
SGITMA	sequential generalized information-theoretic Markov aggregation
GDM	gradient descent method
PIP	performance improvement percentage

## Appendix A

**Proof** **of Lemma 1.** By the definition of the EI,

(A1) $$\begin{array}{cc} EI(P^{\prime })= & I\left(B^{\prime };A^{\prime }|A^{\prime }∼U\right)\\ = & H\left(B^{\prime }\right)-H\left(B^{\prime }|A^{\prime }\right)\\ \leq & H(B^{\prime })\\ \leq & \log(M), \end{array}$$

where the first inequality is due to the nonnegativity of conditional entropy and the last inequality holds because the entropy value of a discrete random quantity is maximized if and only if it is uniformly distributed. From Equation (4), when $P^{\prime }$ is a permutation matrix, there is only one nonzero value in each row and column; thus,

(A2) $$\begin{array}{cc} EI(P^{\prime })= & \sum_{i^{\prime }=1}^{M}\sum_{j^{\prime }=1}^{M}\frac{1}{M}P_{i^{\prime },j^{\prime }}^{\prime }\log P_{i^{\prime },j^{\prime }}^{\prime }-\sum_{j^{\prime }=1}^{M}{\bar{p}}_{j^{\prime }}^{\prime }\log{\bar{p}}_{j^{\prime }}^{\prime }\\ = & \log(M). \end{array}$$

By Equations (A1) and (A2), $EI(P^{\prime })$ is maximized when $P^{\prime }$ is the permutation matrix. Equation (4) can be written as follows:

(A3) $$\begin{array}{cc} EI(P^{\prime }) & =-\sum_{j^{\prime }=1}^{M}{\bar{p}}_{j^{\prime }}^{\prime }\log{\bar{p}}_{j^{\prime }}^{\prime }+\sum_{i^{\prime }=1}^{M}\sum_{j^{\prime }=1}^{M}\frac{1}{M}P_{i^{\prime },j^{\prime }}^{\prime }\log P_{i^{\prime },j^{\prime }}^{\prime }\\ \; & =f_{1}\left(P^{\prime }\right)+f_{2}\left(P^{\prime }\right). \end{array}$$

By the extreme property of entropy,

(A4) $$f_{1}\left(P^{\prime }\right)=-\sum_{j^{\prime }=1}^{M}{\bar{p}}_{j^{\prime }}^{\prime }\log{\bar{p}}_{j^{\prime }}^{\prime }\leq \log\left(M\right),$$

where the equation holds if and only if ${\bar{p}}_{j^{\prime }}^{\prime }=1/M$ holds for any $j^{\prime }$. Then, the column sum of $P^{\prime }$ is unity, which is as follows:

(A5) $$\begin{array}{cc} \frac{1}{M}={\bar{p}}_{j^{\prime }}^{\prime } & =\frac{1}{M}\sum_{i^{\prime }=1}^{M}P_{i^{\prime },j^{\prime }}^{\prime }\\ \; & \Rightarrow \sum_{i^{\prime }=1}^{M}P_{i^{\prime },j^{\prime }}^{\prime }=1. \end{array}$$

As $0\leq P_{i^{\prime },j^{\prime }}^{\prime }\leq 1$, then,

(A6) $$f_{2}\left(P^{\prime }\right)=\sum_{i^{\prime }=1}^{M}\sum_{j^{\prime }=1}^{M}\frac{1}{M}P_{i^{\prime },j^{\prime }}^{\prime }\log P_{i^{\prime },j^{\prime }}^{\prime }\leq 0,$$

where the inequality equal sign holds if and only if

(A7) $$P_{i^{\prime },j^{\prime }}^{\prime }\in \left\{0,1\right\},\forall i^{\prime },j^{\prime }\in X^{\prime }.$$

From Equations (A4) and (A6),

(A8) $$EI\left(P^{\prime }\right)=f_{1}\left(P^{\prime }\right)+f_{2}\left(P^{\prime }\right)\leq \log\left(M\right),$$

We use the method of contradiction to prove that $P^{\prime }$ must be a permutation matrix when $EI(P^{\prime })$ reaches its maximum value. We assume that $EI(P^{\prime })$ can still take its maximum value when $P^{\prime }$ is not a permutation matrix. $f_{1}\left(P^{\prime }\right)=\log\left(M\right),f_{2}\left(P^{\prime }\right)=0$ cannot be held simultaneously, and we discuss the situation separately. When the property of the extreme value for $f_{1}\left(P^{\prime }\right)$ is not satisfied,

(A9) $$f_{1}\left(P^{\prime }\right)<\log\left(M\right),f_{2}\left(P^{\prime }\right)\geq 0$$

By (A3), it can be obtained that

(A10) $$EI\left(P^{\prime }\right)=f_{1}\left(P^{\prime }\right)+f_{2}\left(P^{\prime }\right)<\log\left(M\right)$$

When the nonnegativity of $f_{2}\left(P^{\prime }\right)$ is not satisfied,

(A11) $$f_{1}\left(P^{\prime }\right)\leq \log\left(M\right),f_{2}\left(P^{\prime }\right)>0$$

Then,

(A12) $$EI\left(P^{\prime }\right)=f_{1}\left(P^{\prime }\right)+f_{2}\left(P^{\prime }\right)<\log\left(M\right)$$

From (A10) and (A12), when extreme value and nonnegativity cannot be satisfied simultaneously, it contradicts the assumption that $EI(P^{\prime })$ can still take the maximum value, and the assumption does not hold. Therefore, when $EI(P^{\prime })$ is maximized, $P^{\prime }$ is the permutation matrix. $CE(P^{\prime })$ is also the maximum since there is a fixed difference between $EI(P^{\prime })$ and $CE(P^{\prime })$. □

## Appendix B

**Proof** **of Lemma 2.** The Markov chain is irreducible, which means that for two arbitrary states, one state can reach another state after *m* time steps, while *m* is finite. That is, we need to prove that

(A13) $$\forall a^{\prime },b^{\prime }\in \left[M\right],\exists m^{\prime },P\left\{X_{m^{\prime }}^{\prime }=a^{\prime }|X_{0}^{\prime }=b^{\prime }\right\}>0$$

For brevity, we set $g^{-1}\left(a^{\prime }\right)=\left\{a|W_{a,a^{\prime }}>0\right\}$, $\left\{m_{b,a}\right\}=\left\{l|P\left\{X_{l}=a|X_{0}=b\right\}>0\right\}$, since X is irreducible. Then,

(A14) $$\forall a^{\prime },b^{\prime }\in \left[M\right],\forall a\in g^{-1}\left(a^{\prime }\right),b\in g^{-1}\left(b^{\prime }\right),\exists m_{b,a},P\left\{X_{m_{b,a}}=a|X_{0}=b\right\}>0$$

The set $\left\{m_{b^{\prime },a^{\prime }}^{\prime }\right\}$ is defined as $\left\{m_{b,a}|a\in g^{-1}\left(a^{\prime }\right),b\in g^{-1}\left(b^{\prime }\right)\right\}$; thus, the relationship between $\left\{m_{b,a}\right\}$ and $\left\{m_{b^{\prime },a^{\prime }}^{\prime }\right\}$ is

(A15) $$\left\{m_{b,a}\right\}\subseteq \left\{m_{b^{\prime },a^{\prime }}^{\prime }\right\}$$

From (A14) and (A15), the following can be obtained:

(A16) $$\left.\begin{array}{c} P\{X_{m_{b,a}}=a|X_{0}=b\}>0\\ \left\{m_{b,a}\right\}\in \left\{m_{b^{\prime },a^{\prime }}^{\prime }\right\} \end{array}\right\}⟹\exists l^{\prime }\in \left\{m_{b^{\prime },a^{\prime }}^{\prime }\right\},P\left\{X_{l^{\prime }}^{\prime }=a\prime |X_{0}^{\prime }=b^{\prime }\right\}>0$$

By (A13) and (A16), the irreducibility of $X^{\prime }$ is proven. At time 0, starting from state $a^{\prime }$ and returning to state $a^{\prime }$ after the $m_{a^{\prime }}$ time step, all the time lengths of $a^{\prime }$ belong to the set $\left\{m_{a^{\prime }}:Pr\left(X_{m_{a^{\prime }}}=a^{\prime }|X_{0}=a^{\prime }\right)>0\right\}$. If the greatest common divisor (GCD) of $\left\{m_{a^{\prime }}\right\}$ is 1, then state $a^{\prime }$ is called aperiodic. When all states of $X^{\prime }$ are aperiodic, the Markov chain is aperiodic. We set $g^{-1}\left(b^{\prime }\right)=\left\{b|W_{b,b^{\prime }}>0\right\}$, $\left\{m_{a}\right\}=\left\{l|P\left\{X_{l}=a|X_{0}=a\right\}>0\right\}$; since X is aperiodic,

(A17) $$\forall j\in \left\{g^{-1}\left(b^{\prime }\right)\right\},GCD\left(\left\{m_{j}\right\}\right)=1$$

Since $\left\{m_{b^{\prime }}^{\prime }\right\}=\left\{m_{i,j}|i\in g^{-1}\left(b^{\prime }\right),j\in g^{-1}\left(b^{\prime }\right)\right\}$, then $\left\{m_{j}\right\}\subseteq \left\{m_{b^{\prime }}^{\prime }\right\}$. Therefore, it can be verified that

(A18) $$\left.\begin{array}{c} \left\{m_{j}\right\}\subseteq \left\{m_{b^{\prime }}^{\prime }\right\}\\ GCD\left(\left\{m_{j}\right\}\right)=1 \end{array}\right\}⟹GCD\left(\left\{m_{b^{\prime }}^{\prime }\right\}\right)=1$$

The proof of aperiodicity is complete. From (A16) and (A18), $X^{\prime }$ is also a time-homogeneous, irreducible, aperiodic Markov chain. We use the method of contradiction to prove that $P^{\prime }$ cannot be a permutation matrix. Assuming that W exists, $P^{\prime }$ is a permutation matrix; then, the new Markov chain is periodic, and the cycle number is *M*. However, $X^{\prime }$ is an aperiodic Markov chain, which contradicts the assumption. Therefore, $P^{\prime }$ cannot be a permutation matrix. □

## Appendix C

**Proof** **of Lemma 3.** The nonconvex property can be proven through the following example. Let N=3,M=2,

(A19a)W1=010110,W2=100110,(A19b)W3=12W1+12W2=1/21/20110,(A19c)P1=2/52/51/53/81/43/84/116/111/11,P2=1/41/41/23/51/51/51/31/61/2.

It is easy to verify that

(A20a)CEP1,W3<12CEP1,W1+CEP1,W2(A20b)CEP2,W3>12CEP2,W1+CEP2,W2

Therefore, CE(P,W) is the nonconvex function of W. □

## Appendix D. The Derivation of $\partial_{\beta }CE\left(P,W\right)$

From (8), it can be deduced that

(A21) $${\bar{Q}}_{m^{\prime },n^{\prime }}=\sum_{h=1}^{N}\sum_{l=1}^{N}{\bar{W}}_{h,m^{\prime }}\mu_{h}P_{h,l}{\bar{W}}_{l,n^{\prime }}$$

(A22) $$\begin{array}{cc} \; & \nabla_{\beta }{\bar{Q}}_{m^{\prime },n^{\prime }}\\ \; & =\nabla_{\beta }{\left[\sum_{h=1}^{N}\sum_{l=1}^{N}{\bar{W}}_{h,m^{\prime }}\mu_{h}P_{h,l}{\bar{W}}_{l,n^{\prime }}\right]}_{m^{\prime },n^{\prime }}\\ \; & =-\mu_{i}\frac{W_{i,m^{\prime }}}{1-W_{i,j^{\prime }}}{\left[P\bar{W}\right]}_{i,n^{\prime }}-{\left[{\bar{W}}^{T}\mathrm{diag}\left(\mu \right)P\right]}_{m^{\prime },i}\frac{W_{i,n^{\prime }}}{1-W_{i,j^{\prime }}}\\ \; & +\left\{-\frac{W_{i,m^{\prime }}}{1-W_{i,j^{\prime }}}\mu_{i}P_{i,i}\left(\beta -\left(1-\beta \right)\frac{W_{i,j^{\prime }}}{1-W_{i,j^{\prime }}}\right)+{\left[{\bar{W}}^{T}\mathrm{diag}\left(\mu \right)P\right]}_{m^{\prime },i}\left(1+\frac{W_{i,j^{\prime }}}{1-W_{i,j^{\prime }}}\right)\right\}\left\{\delta \left(n^{\prime }=j^{\prime }\right)\right\}\\ \; & +\left\{-\left(\beta -\left(1-\beta \right)\frac{W_{i,j^{\prime }}}{1-W_{i,j^{\prime }}}\right)\mu_{i}P_{i,i}W_{i,n^{\prime }}+\left(1+\frac{W_{i,j^{\prime }}}{1-W_{i,j^{\prime }}}\right)\mu_{i}{\left[P\bar{W}\right]}_{i,n^{\prime }}\right\}\left\{\delta \left(m^{\prime }=j^{\prime }\right)\right\}\\ \; & +\left\{2\mu_{i}P_{i,i}\left(\beta -\left(1-\beta \right)\frac{W_{i,j^{\prime }}}{1-W_{i,j^{\prime }}}\right)\left(1+\frac{W_{i,j^{\prime }}}{1-W_{i,j^{\prime }}}\right)\right\}\left\{\delta \left(n^{\prime }=j^{\prime }\right)\delta \left(m^{\prime }=j^{\prime }\right)\right\} \end{array}$$

where ${\left[A\right]}_{i,j^{\prime }}$ denotes the $i,j^{\prime }$-th element of matrix A and $\delta \left(a(x)\right)$ is the indicator function with the condition a(x).

From (9), (11), and (12), the following can be obtained:

(A23) $$\begin{array}{cc} \nabla_{\beta }{\bar{P}}_{m^{\prime },n^{\prime }} & =\frac{\left(\sum_{l=1}^{N}{\bar{W}}_{l,m^{\prime }}\mu_{l}\right)\nabla_{\beta }{\bar{Q}}_{m^{\prime },n^{\prime }}-\left\{\delta \left(m^{\prime }=j^{\prime }\right)\mu_{i}-\delta \left(m^{\prime }\neq j^{\prime }\right)\mu_{i}W_{i,m^{\prime }}/\left(1-W_{i,j^{\prime }}\right)\right\}{\bar{Q}}_{m^{\prime },n^{\prime }}}{{\left(\sum_{l=1}^{N}{\bar{W}}_{l,m^{\prime }}\mu_{l}\right)}^{2}}\\ \; & =\frac{{\bar{\mu }}_{m^{\prime }}^{\prime }\nabla_{\beta }{\bar{Q}}_{m^{\prime },n^{\prime }}-\left\{\delta \left(m^{\prime }=j^{\prime }\right)\mu_{i}-\delta \left(m^{\prime }\neq j^{\prime }\right)\mu_{i}W_{i,m^{\prime }}\right\}{\bar{Q}}_{m^{\prime },n^{\prime }}}{{\bar{\mu }}_{m^{\prime }}^{\prime 2}} \end{array}$$

Then, the gradient can be derived by (4), (A22), and (A23):

(A24) $$\begin{array}{cc} \partial_{\beta }CE\left(P,W\right) & =\frac{1}{M}\sum_{m^{\prime }=1}^{M}\sum_{n^{\prime }=1}^{M}\nabla_{\beta }{\bar{P}}_{m^{\prime },n^{\prime }}\left(1+\log{\bar{P}}_{m^{\prime },n^{\prime }}\right)\\ \; & -\sum_{n^{\prime }=1}^{M}\left\{\frac{1}{M}\sum_{m^{\prime }=1}^{M}\nabla_{\beta }{\bar{P}}_{m^{\prime },n^{\prime }}\right\}\left\{1+\log\left(\frac{1}{M}\sum_{m^{\prime }=1}^{M}{\bar{P}}_{m^{\prime },n^{\prime }}\right)\right\} \end{array}$$
